# The role of extra-corporeal radiation therapy for osteo-sarcoma

**DOI:** 10.6026/9732063002001329

**Published:** 2024-10-31

**Authors:** Krutika Mahendra Gohil, Sidharth Misra, Jyothika Venkataswamy Reddy, Shrunga Kandhi Raghavendra, Rishika Singh, Bratish Poddar, Pooja Tawate

**Affiliations:** 1 Department Medicine, Hinduridaysamrat Balasaheb Thackeray Medical College and Dr. Rustom Narsi Cooper Municipal General Hospital, Mumbai, Maharashtra, India; 2 Department Medicine, Armed Forces Medical College, Pune, India; 3 Department of Medicine, Chamarajanagar Institute of Medical Sciences, Chamarajanagar, Karnataka, India; 4 Department of Medicine, Government Medical College and Hospital, Miraj, Maharashtra, India

**Keywords:** Extracorporeal radiation therapy, osteosarcoma, Ewing's sarcoma, Re-implantation

## Abstract

Extracorporeal radiation therapy is a new limb-salvaging procedure increasingly used, especially in situations where it may not be possible to carry out complete surgical resection. Osteosarcoma is a malignant tumour of bone that, typically, presents in young people, and often, it is managed by using different modes of treatment, which include surgery and chemotherapy. This therapy involves the removal of the involved segment of bone, its extracorporeal irradiation, followed by re-implantation to preserve limb function and reduce the risk of recurrence. Therefore, it is of interest to review the effectiveness of, clinical uses, and results of ECRT, focusing on its benefits in terms of bony conservation and decreased complications with either prosthetics or allografts. Promising local control and survival rates further cement its potential.

## Background:

Osteosarcoma is a primary malignant bone tumor, predominantly affecting children and adolescents which are characterized by the production of osteoid by the malignant cells. Osteosarcomas make up for 15% of solid extra cranial cancers with male preponderance [1]. This aggressive cancer is most commonly located in the metaphysis of long bones, such as the distal femur, proximal tibia, and proximal humerus, and areas of rapid bone growth. It is known for its propensity to metastasize to the lungs and presents with pulmonary metastasis at presentation in about 25% patients [2]. It can also occur secondary to Paget disease of Bone or prior radiation exposure. The incidence of osteosarcoma is higher in specific hereditary conditions, like hereditary retinoblastoma and Li-Fraumeni syndrome [3, 4]. Osteosarcoma usually shows a combined inactivation of RB and TP53 pathways. One-fourth of patients show alteration in the PIK3/m TOR pathway [5]. Osteosarcomas do not have distinct tumor markers but elevated levels of lactate dehydrogenase or alkaline phosphatase can be found in a subset of patients. The World Health Organization (WHO) classifies conventional osteosarcoma based on predominant type of matrix within the tumour into osteoplastic, chondroblastic and fibroblastic. The WHO classification recognizes classic, telangiectasia, small cell, periosteal and periosteal, low grade central and high-grade surface osteosarcomas [5]. Treatment spectrum involves surgery, chemotherapy, radiation therapy and supportive care. Despite advances, the 5-year survival rate remains 60% to 78% for localized disease, while metastatic disease presents with a more challenging route [6]. Research into targeted therapies is on-going and yet to yield significant breakthroughs. Due to its heterogeneity, standard treatment protocol is ineffective, necessitating a tailored approach to individual patients. Early detection and a multidisciplinary approach are crucial in improving survival and quality of life.

Extracorporeal radiation therapy (ECRT) is an innovative treatment modality in osteosarcoma management, specifically for cases where complete resection is challenging. Spira and Lubin were the first to document the intra-operative technique of ECRT and re-implantation as an effective strategy for limb salvage in cases of malignant bone tumours [7]. In ECRT, the affected bone segment is surgically excised, treated with high-dose radiation therapy outside the body to eradicate the malignant mesenchyme cells, and then re-implanted. Osteosarcoma, being relatively resistant radiotherapy, benefits from this concentrated, high-dose radiation, which eradicates malignant cells while preserving bone integrity. The goal of ECRT is to enhance local tumor control, reduce risk of recurrence, and improve quality of life, particularly in cases where achieving clear surgical margins is difficult. ECRT and re-implantation is an approach with several benefits, including shorter treatment durations, appropriate graft dimension, elimination of prosthetic wear and tear, and a psychological boost for the patient since their own bone is used for reconstruction. Additionally, this method negates the need for bone banks and minimizes the immunological responses often associated with allografts [8, 9- 10].

## Mechanism of action:

Biopsies are used for Histopathological validation. In order to determine the degree of the disease, the involvement of soft tissue, and the neurovascular bundle, magnetic resonance imaging (MRI) is performed on the affected limb. A positron emission tomography (PET) scan is performed in addition to a chest X-ray to rule out distant metastases and monitor the local extent of the disease. Additionally, a computed CT angiography can be performed on each patient to determine the condition of their vessels [12]. Typically, the patients receive treatment in the order of neoadjuvant chemotherapy, surgery, ECI and re-implantation, and then adjuvant chemotherapy. IAP [Ifosfamide 1.5g/m2 day 1, 2 & 3, Adriamycin 60mg/m2 day 1, and cisplatin 100mg/m2 divided across days 1- 3 regimen is usually administered to osteosarcoma patients every three weeks for six cycles [13]. Four to six weeks later, a broad excision of the afflicted limb in a craniocaudal orientation is performed, leaving a 2 cm margin around the gross tumor. After the bony segment is removed, a complete debridement of the entire tumor and soft tissues is carried out. Subsequently, the bone is wrapped in four layers of sterile drapes, sealed in a plastic bag, and sent for radiation treatment. The surgical site is made ready for re-implantation at the same time. Two parallel opposing anteroposterior-posteroanterior fields are used to deliver a single mid plane dose of 50 Gray to the resected bone [14]. In order to evaluate the condition of the resection margins, biopsy is carried out at each osteotomy site and the operative site is consequently prepared for re-implantation during the ECI. The bone is then re-implanted using fixation devices under C-arm guidance following ECI. Immunisation is maintained throughout the recovery phase until radiographic imaging reveals signs of union. In accordance with radiological and clinical developments, full weight bearing was permitted [13]. The entire procedure is described in Figure 1 - see PDF. The following fifty years have seen comparatively few ECRT procedures. Nonetheless, because limb-sparing procedures have become more common, there has recently been a rise in interest in this orthopaedic oncology method [13]. Moreover, ECI and re-implantation is a biological reconstructive process that has numerous benefits over alternative approaches. In order to protect normal, healthy bodily tissues from radiation damage, the damaged bone fragment is first taken out of the body and exposed to radiation. By using ECI, tumor-bearing bone receives extremely high doses of radiation that would not be conceivable for healthy bone in the body. Second, it is a cost- effective method that offers a graft that is anatomically suited for a biological repair that lasts a lifetime and the maintenance of joint mobility, preventing the issues associated with revision brought on by prosthetic wear. Finally, this method eliminates the requirement for bone banks and a few other allograft-related issues, like graft rejection and the possibility of viral transmission [11].

## Clinical applications:

An important consideration for this technique is to understand the clinical profile of patients who are eligible for this technique. Understanding the selection criteria is crucial to optimize the patient outcomes and minimize complications. The selection criteria include histological diagnosis of malignancy, lack of distant metastasis and suitability for limb preservation [14]. Additionally, tumor location has also been found to be an important determinant for selecting this technique [15]. Patients with structurally weak bones and pathological fractures are not suitable for this technique [16]. Additionally, extensive destruction of the bone by the tumor makes it unsuitable for ERT [17]. The technique of ERT involves administering a dose of 50Gy to the diseased bone segment that has been resected and this dose has been shown to be lethal to all cells and produced a dead auto graft of perfect fit. Also, there is no risk of radiotherapy induced malignancy in the irradiated bone [14]. Though studies are limited by their sample size and lack of heterogeneity, the outcomes of these studies have been promising.

The limb salvage surgery combined with radiation and chemotherapy has become the standard of care for treating bone malignancies. Out of the different subtypes of bone malignancies, ERT has been found to be a feasible option for osteosarcoma, and Ewing's sarcoma. Sharma *et al.* 2020 found this technique to be a feasible option for pediatric bone sarcomas with promising results in terms of good local control and disease-free survival rates [14, 18]. Pruksakorn *et al.* 2018 conducted a retrospective cohort study to assess the surgical outcomes of patients with high grade osteosarcoma who received ERT and re-implantation. The overall failure rate in patients who underwent the intervention was found to be 46% with 40% accounting for non-mechanical failures. In addition, promising outcomes were seen with diaphyseal resection and ERT targeting the intercalary re-implantation region [19]. Davidson *et al.* 2005 treated 50 patients with bony malignancy resection, extracorporeal irradiation with 50 Gy and re-implantation of the bone segment. Of these, 16 had osteosarcomas. After a mean follow-up of 38 months, 42 patients were alive and without disease [20].

Gunaseelan *et al.* 2019 reported their findings on ERT from a tertiary cancer center with good functional outcomes reported in 81.6% of the total patients (40 out of 49). For patients with primary bone malignancies, the local control rate was found to be 94% while the overall complication rate was found to be 20% with infection being characterized as the most common complication (46% of the patients who developed complications) [20]. Furthermore, Hong *et al.* 2013 reported the long-term oncological outcomes of 101 patients from two Australian centers where the main histological diagnoses included Ewing's sarcoma, osteosarcoma, and chondrosarcoma. The 5-year cumulative survival rate was found to be 85.7%. Limb preservation was achieved in 96% of the patients and for those patients with disease affecting the pelvis or lower limb 82.3% had a good functional outcome on their last follow up. Another study conducted by Agarwal *et al.* 2023 analyzed the results of this technique and found a high systemic control rate of 75% at a median follow up period of 12 months [11].

## Advantages of extracorporeal radiation therapy:

Extracorporeal radiation therapy (ERT) offers some notable advantages when it comes to treating bone tumors that are worth mentioning. With a primary aim of preserving bone stock, it prevents the transmission of the disease and preserves limb function [21]. This technique mainly involves the removal of the tumor bearing bone followed by irradiation and re-implantation of the bone [14]. It has been found to be appropriate for various tumor locations such as femur, tibia, and humerus with osteosarcoma being the most common tumor histological subtype [18, 20]. ERT is particularly beneficial for patients who have an immature skeleton and research suggests an average union time of 8.1 months. It has proven to be a cost-effective treatment option when compared to artificial prostheses [17]. Studies have also reported good local control rates of 73-94% and satisfactory functional outcomes with mean Musculoskeletal Tumor Society scores ranging from 26 to 88 [14, 22, 24]. Additionally, this technique has been found to be effective in diaphyseal resections and intercalary re-implantation with fewer structural complications and biological permanence [19]. The outcomes of ERT in pelvic bone tumors and reported functional scores averaging 70% on the Enneking scale for patients treated with this technique [23]. It is crucial to understand the differences between extracorporeal radiation therapy and other conventional radiation techniques. The technique of extracorporeal radiation eliminates the risk of disease transmission and the immunological reactions associated with other conventional radiation techniques [21]. There is an added advantage of using the patient's own bone which ensures easier anatomical re-attachment of muscles and tendons [8, 10]. This in turn ensures preservation of natural joint anatomy and mobility. Consequently, it also eliminates the problems associated with artificial prostheses. Davidson *et al.* 2005 studied the use of extracorporeal irradiation on patients who underwent en-bloc resection for bone malignancies and found reliable union of bone osteotomies [9]. Promising results have been seen in local control and short-term survival rates with Sharma *et al.* 2013 reporting a 2-year local recurrence free survival rate of 73% [14]. Another study conducted by Gunaseelan *et al.* in 2019 reported the 8-year local recurrence free and distant metastasis free survival rates to be 89% and 84%, respectively [20].

With efforts to improve the management of bone malignancies, there has been an increase in the integration of extracorporeal radiation therapy into clinical practice. Increased precision, maintenance of limb functionality and the use of patient's own bone are key hallmark features associated with this technique [13, 20]. In addition, using the patient's native bone reduces the risk of complications that are associated with the use of foreign implants such as infection and implant failure [24]. Considering these advantages, it is quintessential to incorporate extracorporeal radiation technique in the multi-disciplinary management of bone malignancies.

## Future directions:

## Recommendations for subsequent research:

Future research should focus on long-term effects of extracorporeal irradiation (ECI), with larger sample sizes and standardized dosages to assess survival and functional outcomes [10, 25]. Customized radiation dosing for improved limb salvage outcomes, especially in younger patients, warrants investigation [26, 27, 28, 29-30]. Comparative studies on ECI versus prostheses and allografts are needed [9, 26]. Integration of biomarkers may personalize treatment [10, 26 and 28]. Advancements in technology like IMRT, IGRT, 3D-printed prosthetics, and nanotechnology could refine radiation delivery and bone healing [25, 14, and 16]. Multidisciplinary approaches optimize patient care from diagnosis to rehabilitation, emphasizing individualized, team-based strategies [27, 28, 10, 16 and 14].

## Limitations:

Extracorporeal irradiation followed by re-implantation of irradiated auto-grafts can lead to complications such as local recurrence, delayed healing of the graft-host junction, osteochondral collapse, and fixative device failure. These complications are difficult to compare due to factors such as tumor type, sterilization techniques, reconstruction site, and chemotherapy status. Failures are primarily categorized as "mechanical failure" and "non-mechanical failure [11, 31-32].

## Conclusion:

For patients with bone cancers, ECRT has become a viable option for limb salvage. Research has indicated that it is efficacious in maintaining limb function and providing satisfactory local control, particularly in cases of osteosarcoma and Ewing's sarcoma. ECRT has demonstrated good percentages of disease-free survival and functionality when patients are carefully chosen based on the kind of tumor, location, and degree of bone loss.
